# Pupation Substrate Type and Volume Affect Pupation, Quality Parameters and Production Costs of a Reproductive Colony of *Ceratitis capitata* (Diptera: Tephritidae) VIENNA 8 Genetic Sexing Strain

**DOI:** 10.3390/insects12040337

**Published:** 2021-04-09

**Authors:** Carlos Pascacio-Villafán, Luis Quintero-Fong, Larissa Guillén, José Pedro Rivera-Ciprian, Reynaldo Aguilar, Martín Aluja

**Affiliations:** 1 Instituto de Ecología, A.C. (INECOL), Clúster Científico y Tecnológico BioMimic®, Red de Manejo Biorracional de Plagas y Vectores, Xalapa 91073, Veracruz, Mexico; larissa.guillen@inecol.mx (L.G.); martin.aluja@inecol.mx (M.A.); 2 Programa Operativo Moscamed-México, Acuerdo SAGARPA-IICA, Subdirección de Validación y Desarrollo Tecnológico, Carretera Tapachula Cd. Hidalgo km 19.5 S/N., Metapa de Domínguez CP. 30860, Chiapas, Mexico; jose.rivera.i@senasica.gob.mx (J.P.R.-C.); reynaldo.aguilar.i@senasica.gob.mx (R.A.)

**Keywords:** female pupal weight, sterile insect technique, Mediterranean fruit fly, mass-rearing, mother colony, cellulose

## Abstract

**Simple Summary:**

The Mediterranean fruit fly, *Ceratitis capitata*, is a pest of fruit and vegetables in many parts of the world. The Sterile Insect Technique (SIT) is used as an environmentally friendly method for the control and/or eradication of this pest. A critical step in the production of sterile insects in biofactories is when the larvae complete their development and begin metamorphosis to become pupae. Artificial substrates are used to facilitate pupation, but little research has been devoted to this critical moment in the life cycle of the insect. We tested seven pupation substrates and five substrate volumes for a reproductive colony of *C. capitata* VIENNA 8 genetic sexing strain for SIT application in Mexico. One type of cellulose and sawdust at relatively low ratios of substrate:larvae enhanced pupation and were more cost-effective to produce one million pupae than other substrates including fine wheat bran currently used in a SIT mass-rearing facility in Mexico. Our study contributes to improving rearing processes involved in SIT-based pest control.

**Abstract:**

Adequate pupation substrates and substrate volume are critical factors in the mass-rearing of insects for Sterile Insect Technique (SIT) applications. To identify an ideal pupation substrate for a reproductive colony of *Ceratitis capitata* (Wiedemann) VIENNA 8 genetic sexing strain, we first examined pupation in cellulose from recycled paper (cellulose I), sawdust, fine wheat bran, vermiculite and coconut fiber using a volume of 2.5–12.5 mL of substrate for each 5 mL volume of fly larvae. We found a positive relationship between substrate volume and pupation, with cellulose I generating the highest proportions of pupation and coconut fiber the lowest. Higher proportions of female flies (white pupae) pupated in sawdust. The proportion of female fliers increased as substrate volume rose in sawdust and coconut fiber, whereas it decreased in vermiculite and cellulose. In a second experiment, we tested three types of cellulose differing in physicochemical characteristics (celluloses I, II and III), sawdust, and fine wheat bran using a substrate:larvae ratio of 1:1. The three types of cellulose produced the highest pupation levels. The highest proportions of female fliers were observed in sawdust, and cellulose types III and II. Cellulose III and sawdust at relatively low volumes were more cost-effective to produce one million pupae than other substrates, including fine wheat bran used in a mass-rearing facility in Mexico.

## 1. Introduction

The Mediterranean fruit fly, *Ceratitis capitata* (Wiedemann) (Diptera: Tephritidae), is a highly polyphagous species native to Africa that has invaded most tropical and subtropical regions of the world [1,2]. *Ceratitis capitata* attacks the fruit of more than 300 wild and cultivated plant species and is considered one of the most serious pests in fruit and vegetable industries worldwide [3,4,5]. The Sterile Insect Technique (SIT) is used in many countries as an effective and environmentally friendly method of pest control against *C. capitata* [6,7,8,9].

In Mexico, the Moscamed mass-rearing facility, in Metapa de Domínguez, Chiapas, produces an average of 500 million male pupae of *C. capitata* VIENNA 8 genetic sexing strain (GSS) per week for SIT releases [7,10]. The VIENNA 8 GSS is based on the white pupae (wp) and temperature sensitive lethal (tsl) mutations [11]. These mutations result in the death of female insects following high-temperature treatment (34 °C) during the egg stage and allow separation of pupae by color (female and male pupae have white and brown puparia, respectively) [12,13,14]. The production of sterile male *C. capitata* VIENNA 8 GSS in Mexico is expected to reach one billion flies per week once a recently constructed mass-rearing facility is fully operational [15]. The production of sterile flies in the new Moscamed facility, will be based on a colony of reproductive flies (i.e., a mother colony with female and male flies) from which eggs will be obtained [16]. This reproductive colony is currently maintained on a larval artificial diet from which third instar larvae that are ready to pupate fall into trays with water, larvae are then collected and placed in fine wheat bran to eliminate excess moisture for insect pupation [16]. In nature, *C. capitata* larvae also leave their host fruit once they have completed their development to burrow into the ground and pupate [17].

As in nature, pupation of flies in a mass-rearing context is critical, because during metamorphosis insects are exposed to several factors that affect their development, survival, and quality for SIT purposes [18,19]. For instance, Langley and collaborators [20], working with *C. capitata*, found that the rate of water loss was greatest during the first three days of pupal formation and that pupae formed optimally when air temperature and relative humidity in the pupation room varied between 20–25 °C and 75–90% RH, respectively. Vargas et al. [21], also working with *C. capitata*, found that mean pupal weight, and adult eclosion and fecundity were significantly higher for larvae that pupated in vermiculite when compared to those that pupated without vermiculite, i.e., naked larvae. Importantly, the lack of a pupation medium significantly reduced adult survivorship. For example, 14 days after emergence, 35.4% of adults that stemmed from “naked larvae”, had already died, whereas only 12% adults stemming from larvae that pupated in adequate pupation conditions had perished. These authors indicated that the physical proximity of naked larvae and the metabolic heat they produced while moving around in search of an adequate pupation site, significantly reduced the quality of pupae. They concluded that “the use of moist vermiculite as a pupation medium not only absorbs heat but also spatially separates larvae”, highlighting the need to identify optimal pupation conditions [21].

Pupation substrates that have been used or tested for artificial rearing of tephritid fruit flies include sawdust, wheat bran, rice bran, vermiculite, coconut fiber, sand, organic soil (sandy, loamy) and corncob grits, among others [19,21,22,23,24,25,26,27,28]. Fruit fly pupation differs as a function of the substrate used as shown by Celedonio et al. [23], who tested organic soil, sand mixed with organic soil, sand, vermiculite, vermiculite mixed with soil or sand, and pupation in the absence of a substrate (i.e., naked pupation) with *Anastrepha serpentina* (Wiedemann). They found that organic soil was the best substrate with 90% of larvae pupating, followed by sand mixed with organic soil (75%), sand alone (73%), vermiculite and soil (72%), vermiculite alone (65%) and vermiculite mixed with sand (58.3%). In the case of pupation in the absence of a substrate the recovery rate was just 5%. Sookar et al. [27] tested two pupation substrates (dry/moistened sand and wetted sand) for *Bactrocera zonata* (Saunders) and compared them with the “naked-larvae” pupation system and following FAO/IAEA/USDA [29] guidelines assessed pupal weight, adult emergence and volume of pupae produced. Notably, naked larvae pupated three days after larvae provided with a pupation medium which pupated within 24 h. Pupae stemming from sand were significantly heavier than those obtained from vermiculite. Pupae formed in moistened media were significantly heavier than pupae obtained from dry media. Pupation media had a significant effect on the proportion of larvae that transformed into pupae with 82.0, 78.4 and 65.1% of pupation in vermiculite, sand and in the naked condition, respectively. Nevertheless, pupation substrate did not influence adult emergence and proportion fliers.

Despite the relevance of pupal well-being in mass-rearing of tephritid fruit flies, research on the topic of pupation substrates for *C. capitata* is relatively scant compared to other areas of research related to the mass-rearing of this fly pest for SIT applications (but see [21,30]). In line with the philosophy of the new Moscamed-Mexico facility to use science-based understanding in the decision-making process related to the mass rearing of *C. capitata*, as well as the permanent goal of optimizing rearing cost through innovation in any phase of insect production [16], here we tested seven pupation substrates and five substrate volumes for a reproductive colony of *C. capitata* VIENNA 8 GSS to identify a substrate that could provide a cost-effective alternative to the fine wheat bran substrate currently in use. One downside of the fine wheat bran substrate is that when it gets wet it forms lumps that adhere to the pupating larvae hindering pupal processing. We focused on female flies, as this is the most valued sex in the rearing process of reproductive colonies of *C. capitata* VIENNA 8 GSS carrying the tsl mutation.

## 2. Materials and Methods

### 2.1. Insects

Experimental larvae were obtained from a reproductive colony of *C. capitata* VIENNA 8 GSS maintained at the Moscamed-Mexico mass-rearing facility in Metapa de Domínguez, Chiapas, Mexico. This colony of *C. capitata* VIENNA 8 GSS was originally provided by the El Pino mass-rearing facility in Barberena, Guatemala. The colony had been reared for 17 generations in the Moscamed-Mexico facility when this study was initiated. Adult flies were kept in cages (51 cm height × 51 cm width × 84 cm length), lined with metal mesh, at a density of 22,000 flies per cage (ca. 12,000 females and 10,000 males). Flies were provided ad libitum access to food (sugar and hydrolyzed protein in a 3:1 ratio) and water inside cages. Female flies oviposited in oviposition panels placed at the sides of the cages. Eggs were collected from these panels and incubated in aerated water (15–30 mL of eggs/2 L of water) at 24 °C for 48 h. Then, a volume of ca. 1.0 mL of eggs in a guar gum solution was inoculated into 1 kg of artificial diet and larvae were reared following the starter-finalizer larval diet system for 17 days at 24 °C, 75 ± 5% relative humidity (RH) and 12:12 h L:D photoperiod [16]. When larvae reached the third instar and were ready to pupate, they left the diet and fell into aluminum trays with water, from which they were retrieved [16]. Each batch of diet yielded nine batches of larvae that were harvested over nine consecutive days. In the experiments, we used larvae from the fifth (Experiment 1) and sixth (Experiment 2) batches. After retrieval from water, larvae were dried with fine wheat bran, then sieved to remove all the bran and used in the experiments.

### 2.2. Pupation Substrates

We tested fine wheat bran, vermiculite, sawdust, coconut fiber and three types of cellulose (referred to as celluloses I, II and III) as pupation substrates differing in their cost per kg and physicochemical characteristics (Table 1). Only the sawdust was sifted before use in the experiments to separate big chips that could injure the larvae. Physicochemical characteristics of the substrates were determined at the Quality Control Laboratory of the Moscamed facility following standard methods [31,32,33]. Fine wheat bran is the pupation substrate currently used for *C. capitata* VIENNA 8 GSS at the Moscamed facility; vermiculite and sawdust are substrates reported elsewhere for pupation of *C. capitata* and other tephritids [21,23]; coconut fiber has not been previously tested with *C. capitata* and was of interest because of initial promising results as a pupation substrate for the West Indian fruit fly (i.e., mango fruit fly), *Anastrepha obliqua* (Macquart) [19]; cellulose has not been reported as a pupation substrate for tephritid fruit flies, and was of interest because of its fine texture and preliminary positive observations in the form of cellulose I as a pupation substrate for the male-only rearing (R. Aguilar personal observation).

### 2.3. Experimental Design

#### 2.3.1. Experiment 1

The explanatory variables were: (i) the volume of pupation substrate in a continuous scale (2.5, 5, 7.5, 10 and 12.5 mL of substrate per 5 mL of third instar *C. capitata* VIENNA 8 GSS larvae from the reproductive colony), and (ii) the type of pupation substrate (cellulose I, sawdust, fine wheat bran, vermiculite and coconut fiber). The response variables were: (i) the proportion of larvae that pupated (i.e., pupation of females and males), (ii) sex ratio of pupae (proportion of female pupae), (iii) female emergence (proportion), and (iv) female fliers (proportion). Details on how these variables were measured are presented in Section 2.4. (Experimental Protocol). The 5 mL volume of larvae was equivalent to a mean (± standard error) of 267.3 ± 3.7 third instar larvae (*n* = 50). The range of substrate volumes tested (2.5 to 12.5 mL of substrate per 5 mL of larvae), was equivalent to a substrate:larvae ratio ranging from half a part of substrate:one part of larvae (0.5:1) to 2.5 parts of substrate:one part of larvae (2.5:1). This range of substrate:larvae ratios was designed to include the 1:1 ratio used in the reproductive colony of *C. capitata* VIENNA 8 GSS at the Moscamed facility, and to test a contrasting range of substrate volumes from low to high. The experiment consisted of 25 combinations of substrate type × substrate volume, each replicated two times for a total of 50 experimental runs. The experimental unit comprised a Petri dish (15 cm diameter × 2.5 cm tall) with 2.5–12.5 mL of substrate. The substrate type × substrate volume combinations were assigned randomly to the experimental units.

#### 2.3.2. Experiment 2

The explanatory variable was the type of pupation substrate (celluloses I, II and III, sawdust and fine wheat bran); and the response variables were: (i) the proportion of larvae that pupated (i.e., pupation of females and males), (ii) female pupal weight (mg), (iii) female emergence (proportion), and (iv) female fliers (proportion). Details on how these variables were measured are presented in the following Section 2.4. (Experimental protocol). The experimental unit comprised a Petri dish (15 cm diameter × 2.5 cm tall) with 20 mL of a specific pupation substrate and 20 mL of larvae. This substrate:larvae ratio is equivalent to the 1:1 ratio normally used in the reproductive colony at the Moscamed facility. The experiment was performed as a randomized block design, with each type of pupation substrate replicated 15 times for a total of 75 experimental units divided among five blocks over time (days).

### 2.4. Experimental Protocol

All the experimental procedures were performed at the Moscamed facility, in Metapa de Domínguez, Chiapas, Mexico.

#### 2.4.1. Experiment 1

Third instar *C. capitata* VIENNA 8 GSS larvae were obtained as indicated in Section 2.1. Dry larvae and pupation substrates were measured with a graduated cylinder and deposited in Petri dishes (15 cm diameter × 2.5 cm tall). We used a fixed volume of 5 mL of third-instar *C. capitata* VIENNA 8 GSS larvae with varying volumes and types of pupation substrates as indicated in Section 2.3.1. The Petri dishes with substrate and larvae were closed with a lid and placed in a pupation laboratory at 24 °C ± 1 °C, 75 ± 5% RH in constant darkness. After 24 h, the number of larvae and pupae per Petri dish was recorded and pupation was estimated as the number of pupae (female and male) divided by the total number of individuals per Petri dish (i.e., larvae that had not yet pupated and pupae). Larvae that had not pupated at this point were not considered further in the experiment. Petri dishes with substrate and pupae were maintained in the pupation laboratory for a total of five days. The pupae were then separated from the substrate by sieving and returned to their respective Petri dish. The Petri dishes with pupae and without substrate were incubated for six additional days at 21 °C, 75–85% RH and photoperiod of 12:12 h L:D. Then, the number of female and male pupae was recorded, and the non-viable pupae (e.g., black pupae) were separated. The sex ratio of pupae (expressed as the proportion of female pupae) per Petri dish was estimated as the number of female pupae (white pupae) divided by the total number of pupae present. Samples of female pupae (50–86 pupae) were further used to test emergence and flight ability [34]. Pupae were placed inside black PVC cylinders (10 cm height × 8.9 cm diameter) coated with unscented talcum powder in the interior. Tubes with pupae were placed inside metal cages (150 cm height × 130 width × 60 cm depth) lined with an anti-aphid mesh in a laboratory at 25 ± 1 °C, 70 ± 5% RH and a 12 h:12 h L:D photoperiod. Flies emerged inside the tubes and the individuals capable of flying (i.e., fliers) exited the tubes. Fliers were removed daily from test cages. After four days, the remaining contents of the tubes (i.e., empty puparia, non-emerged pupae, and non-flier adult females) were counted. The proportion of emergence per tube was calculated from the number of empty puparia (i.e., individuals that emerged) and the number of pupae placed initially inside the tube [34]. The proportion of fliers per tube was calculated from the number of flies that flew out the tube (i.e., empty puparia minus adult flies that remained inside the tube) and the total number of pupae in the tube [34].

#### 2.4.2. Experiment 2

Larvae were obtained as indicated in Section 2.1. We used 20 mL of larvae with 20 mL of a specific pupation substrate. Petri dishes with substrate and pupae were treated in the same way as in Experiment 1. Pupation was estimated as in Experiment 1. Mean female pupal weight was estimated by weighing samples of 100 11-day-old pupae per experimental unit on an electronic balance (A&D-HR-250AZ, A&D Co. Ltd., Tokyo, Japan). These pupae were also used to test emergence and flight ability as indicated in Experiment 1 (Section 2.4.1).

### 2.5. Statistical Analyses

#### 2.5.1. Experiment 1

We used analysis of covariance with a generalized linear model (GLM) approach to model the data of each response variable as a function of categorical (pupation substrates) and continuous (substrate volumes) explanatory variables. GLMs with quasibinomial error distribution and logit link function were fitted to our data as these were proportions estimated from samples of varying sizes (50 to 86 pupae in the cases of female emergence and female fliers) that exhibited overdispersion after fitting binomial GLMs [35]. Models were fitted to the data of each response variable considering main and interaction effects of the explanatory variables. In the case of pupal sex ratio, female emergence and female fliers the observations of two runs (out of a total of 50) were missed from the database due to logistical reasons. Significant effects (*p*-value < 0.05) were assessed by analysis of deviance with an *F* test [35]. When non-significant model terms were found (*p*-value ≥ 0.05), model simplification was performed by removing non-significant model terms beginning with the interaction term [36]. Every time a term was removed from a model, analysis of deviance with an *F* distribution was applied to test if the removal of terms significantly affected the explanatory power of the model. If removing a term from a model caused a significant increase in deviance, then the term was retained in the model [36]. The goal was to identify the minimum significant model to describe our data [36]. Assumptions of normality and homoscedasticity were confirmed graphically by examination of the model residuals. Analyses were performed using the software R [37].

#### 2.5.2. Experiment 2

We used analysis of variance (ANOVA) to test the null hypothesis that the mean values of the response variables evaluated did not differ among the type of pupation substrate tested. Data on pupation, female emergence, and female fliers (i.e., proportional data estimated from sample sizes of *n* = 100) were logit transformed [38]. When significant effects were detected by ANOVA (*p*-value < 0.05), multiple comparisons of means were performed. Assumptions of normality and homoscedasticity were confirmed graphically with the model residuals. Analyses were performed with the software R [37] and the “multcomp” package [39].

#### 2.5.3. Estimation of Quantities of Larvae and Costs of Substrates Required to Produce One Million Pupae

Based on the analyses of pupation from Experiments 1 and 2, on the cost per kg and density of pupation substrates (Table 1), and on a mean number of *C. capitata* VIENNA 8 GSS third instar larvae in a 5 mL volume (267.3 larvae), we estimated the quantities of third instar larvae and costs of the pupation substrates at selected volumes required to produce one million pupae of *C. capitata* VIENNA 8 GSS. In the case of celluloses I, II and III, we selected the lowest volume tested (2.5 mL of substrate per 5 mL of larvae), because in Experiment 1, cellulose I proved to be the substrate with the highest proportions of pupation, even at the lowest volume of substrate and, in Experiment 2, pupation did not differ significantly among the three celluloses. In the case of fine wheat bran, sawdust, and vermiculite, we selected the volume of 5 mL of substrate per 5 mL of larvae (i.e., one part of substrate per one part of larvae), as this substrate:volume ratio was used for the reproductive colony of *C. capitata* VIENNA 8 GSS in the Moscamed facility, with high and similar proportions of pupation in the three substrates. In the case of coconut fiber, the volume of 12.5 mL of substrate/5 mL of larvae was selected, because this substrate produced overall low pupation compared to the other substrates and the selected volume resulted in the highest proportion of pupation.

The number of larvae required to produce one million pupae on each substrate was estimated as: one million pupae divided by the predicted proportion of pupation of the quasibinomial GLM model for each substrate and volume selected. The volumes selected were: 2.5 mL of substrate per 5 mL of larvae in the case of cellulose I (this volume was also used for celluloses II and III); 5 mL of substrate per 5 mL of larvae in the case of fine wheat bran, sawdust, and vermiculite; and 12.5 mL of substrate per 5 mL of larvae in the case of coconut fiber. These volumes of substrate per 5 mL of larvae are equivalent to substrate:larvae ratios of 0.5:1 (for celluloses I, II and III), 1:1 (for fine wheat bran, sawdust, and vermiculite) and 2.5:1 (for coconut fiber).

The cost (in US $) of substrates required to produce one million pupae was estimated as: the weight (kg) of substrate volume needed to produce one million pupae × the cost of substrate per kg (US $) (Table 1).

The weight (kg) of the specific substrate volume required to produce one million pupae was estimated as: (substrate density (Table 1) × substrate volume (mL) required to produce 1 million pupae)/1000 g.

The volume (mL) of substrate required to produce one million pupae was estimated as: [(Number of larvae required to produce one million pupae × 5 mL of larvae)/267.3 larvae in 5 mL] × Selected substrate volume/5 mL of larvae.

## 3. Results

### 3.1. Experiment 1

**Figure 1:**
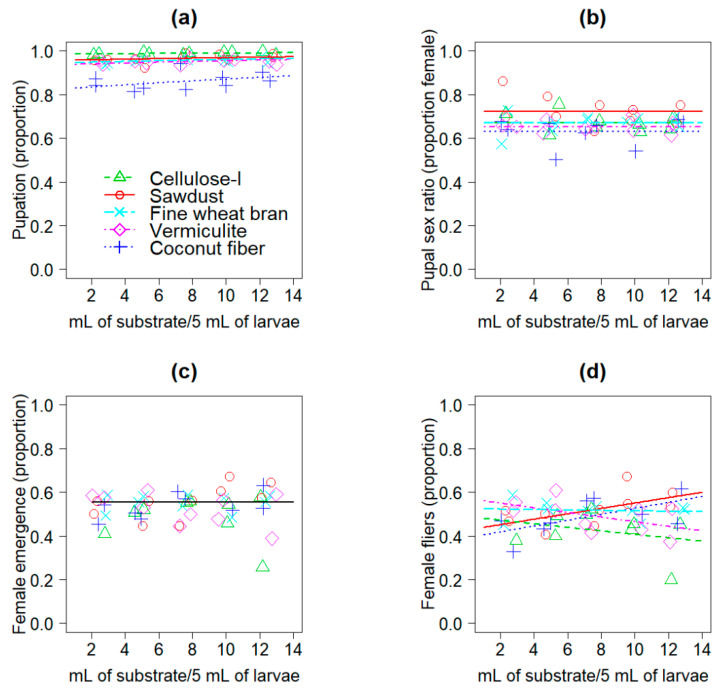
Models fitted to data on (**a**) pupation, (**b**) pupal sex ratio, (**c**) female emergence and (**d**) female fliers of *C. capitata* VIENNA 8 GSS as a function of the type of pupation substrate and substrate volume with a fixed volume of larvae (5 mL). Lines represent the models and symbols jittered data points; in (**c**) the horizontal black line represents the null model.

The proportion of *C. capitata* VIENNA 8 GSS larvae that pupated differed significantly as a function of the substrate type (*F*_4, 45_ = 64.9, *p* < 0.0001) and substrate volume (*F*_1, 44_ = 6.5, *p* = 0.0142). Substrate volume had a positive effect on pupation (model estimate = 0.036, *t* = 2.56, *p* = 0.0144, Supplementary Materials File S1; Figure 1a). Cellulose I yielded the highest proportions of pupation and coconut fiber the lowest, whereas pupation in sawdust, vermiculite and fine wheat bran was similar (Figure 1a). Sex ratio of pupae differed significantly only as a function of the type of substrate used (*F*_4, 43_ = 4.19, *p* = 0.0059); the highest proportions of females that pupated (i.e., white pupae) were observed in sawdust (0.723), followed by fine wheat bran (0.672) and cellulose I (0.672), vermiculite (0.653), and coconut fiber (0.631) with the lowest proportion of female pupation (Figure 1b). In the case of female emergence, we did not find a significant effect of substrate type (*F*_4, 43_ = 1.51, *p* = 0.2191), volume (*F*_1, 42_ = 0.48, *p* = 0.4937), or their interaction (*F*_4, 38_ = 1.97, *p* = 0.1179), and the model simplification led to a null model indicating that an overall mean proportion of 0.533 provided the best description of female emergence (Figure 1c). A significant interaction between substrate type and volume affected the proportion of female fliers (*F*_4, 38_ = 3.0, *p* = 0.0305); the proportion of female fliers increased as a function of the volume of substrate only in the case of sawdust and coconut fiber, whereas in the case of cellulose and vermiculite the proportion of female fliers decreased as a function of the volume of substrate used, and in the case of fine wheat bran the proportion of female fliers was similar across the range of substrate volumes used (Figure 1d). The linear predictors of the models fitted to the data of the different response variables are presented in Supplementary Materials File S1.

### 3.2. Experiment 2

**Figure 2:**
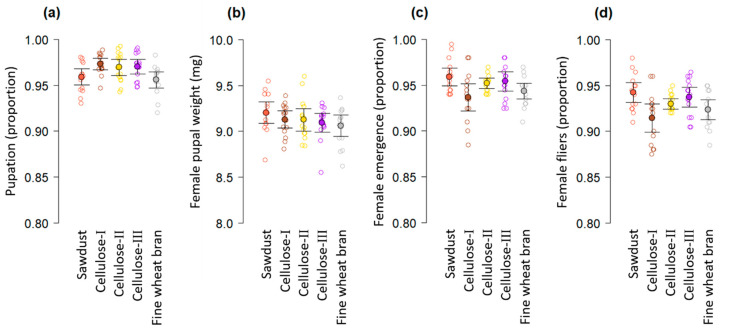
Mean of (**a**) pupation, (**b**) female pupal weight, (**c**) female emergence (proportion) and (**d**) female fliers (proportion) of *C. capitata* VIENNA 8 GSS as a function of the type of pupation substrate. Open symbols indicate jittered data points, solid symbols average values and the error bars indicate 95% confidence intervals.

The mean proportion of pupation exceeded 0.95 in all substrates, but there were significant differences in pupation as a function of the type of substrate used after controlling for block effects (*F*_4, 68_ = 3.91, *p* = 0.0064). The highest proportions of pupation were observed with the three types of cellulose and the lowest with sawdust and fine wheat bran (Figure 2a), but a significant difference was only detected between cellulose I and fine wheat bran (*t* = 3.01, *p* = 0.0291; Supplementary Materials File S2). The weight of 11-day-old female pupae did not differ significantly according to the type of pupation substrate used (*F*_4, 68_ = 1.57, *p* = 0.1935), and the mean pupal weight was more than 9 mg in all cases (Figure 2b). The proportion of female emergence (*F*_4, 68_ = 2.8, *p* = 0.0325, Figure 2c) and female fliers (*F*_4, 68_ = 4.18, *p* = 0.0044, Figure 2d) differed significantly as a function of the type of pupation substrate used, following a similar trend, with the highest mean proportions observed in larvae that pupated in sawdust, followed by cellulose III and cellulose II, whereas the lowest proportions were observed in fine wheat bran and cellulose I. However, the only significant difference in the proportion of female emergence and female fliers was observed between cellulose I and sawdust (female emergence: *t* = 2.93, *p* = 0.0356; female fliers: *t* = 3.61, *p* = 0.0051; Supplementary Materials File S2).

### 3.3. Estimation of Quantities of Larvae and Costs of Substrates Required to Produce One Million Pupae

The lowest number of larvae for the production of one million pupae was estimated for celluloses I, II and III, whereas the highest was estimated for coconut fiber (Table 2). The number of larvae estimated to produce one million pupae in fine wheat bran was higher than in celluloses I, II and III, and sawdust, but lower than in vermiculite and coconut fiber (Table 2). Cellulose I was by far the most economical substrate for producing one million pupae of *C. capitata* VIENNA 8 GSS, followed by coconut fiber and sawdust with a cost of more than double that of cellulose I (Table 2). Cellulose III was more economical to produce one million pupae than fine wheat bran, vermiculite, and cellulose II (Table 2), but was more expensive than sawdust (Table 2). Cellulose II was the most expensive substrate to produce one million pupae (Table 2).

## 4. Discussion

Although mass rearing of fruit flies and particularly of *C. capitata* has a long and illustrious history in more than 10 countries around the world [8,9,22], there are still ways that rearing protocols of this species can be improved. It was clear from our results that the type of substrate and the substrate to larvae ratio are critical factors that affected the pupation and quality of a reproductive colony of *C. capitata* VIENNA 8 GSS (Figure 1 and Figure 2). The positive effects of substrate volume on pupation (Figure 1a), reflect the fact that pupation is when metamorphosis takes place, which is the most energy demanding process in the life cycle of holometabolous insects [40,41,42]. The stress and energetic investment of a larva that is unable to naturally bury itself in substrate to pupate may be high which could potentially disrupt pupation. In fact, the metabolic heat of wandering larvae while moving around in search of an adequate pupation site can reduce the quality of pupae of mass-reared *C. capitata* [21]. Having a pupation substrate that produces a high proportion of pupation with the smallest quantity of substrate is highly attractive in a mass-rearing context to save resources, reduce pupation waste, and facilitate daily handling of pupation materials. With this in mind, we note that even relatively low volumes of cellulose substrates produced the highest pupation of a reproductive colony of the *C. capitata* VIENNA 8 GSS compared to other substrates, including the fine wheat bran that is currently used in the Moscamed facility (Figure 1a and Figure 2a). Fine wheat bran was the second most economical substrate per kg (Table 1), but cellulose I, coconut fiber, sawdust and cellulose III were estimated to be more economical than fine wheat bran for the production of one million pupae (Table 2). In part, this was because the celluloses, coconut fiber and sawdust had lower densities than fine wheat bran (Table 1) allowing a reduction in the weight of substrate used and associated cost (Table 2). In what follows, we will discuss our findings and relate them to previous published studies on the topic, which are surprisingly scant given the economic relevance of pupal well-being in mass-rearing facilities of sterile insects for SIT applications.

Pupation of *C. capitata* in a mass-rearing context was previously studied by Vargas et al. [21], who proved the benefits of using pupation substrates in the quality of artificially reared flies compared to flies that pupated in the absence of any substrate. Conversely, Hooper [43] argued in favor of naked pupation over pupation in substrate when larvae were collected in rotary tumblers. The naked pupation system is a common practice in many mass-rearing facilities as an inexpensive alternative and is the current method used for the male-only rearing at the Moscamed facility. However, rearing of female flies of *C. capitata* VIENNA 8 GSS in the Moscamed facility require different protocols, including the use of a substrate to reduce the risk of damage to female flies as naked larvae tend to stick to the surface they are crawling on or stick to neighboring larvae/pupae, causing damage to the integument (R. Aguilar personal observation). Our study did not include a pupation treatment in the absence of a substrate, but instead we tested several pupation substrates and substrate to larvae ratios in a single study. Notably, this is the first report on the use of cellulose and coconut fiber as pupation substrates for a reproductive colony of *C. capitata* VIENNA 8 GSS. The three types of cellulose we tested yielded the highest proportions of pupation (Figure 1a and Figure 2a), but cellulose III produced higher proportions of female fliers than celluloses I and II (Figure 2d). Sawdust also produced higher proportions of female fliers than cellulose I (Figure 2d). In contrast to cellulose I, the coconut fiber substrate produced the lowest proportions of pupation (Figure 1a), and was the substrate requiring the largest number of larvae to produce one million pupae according to our analyses (Table 2). This disadvantage of coconut fiber compared to cellulose pupation substrates means that a relatively large number of larvae would be lost in coconut fiber due to the low proportion of pupation in this substrate (Figure 1a, Table 2). Future studies should estimate the cost of larvae that do not pupate and die due to the lack of an adequate substrate, to generate more precise information on production costs. Sawdust was US $0.24 more economical than cellulose III for producing one million pupae, but the predicted proportion of pupation in sawdust was 0.024 lower than in cellulose III (Table 2). This translates into a loss of ca. 25,000 larvae per million pupae produced in sawdust compared to cellulose III. However, this could be overcome by the fact that sawdust was the substrate that produced the highest proportion of female pupae (Figure 1b). Having high proportions of female fliers as observed in our study with sawdust and cellulose III (Figure 2d), could be important for oviposition rates if females must fly to reach the oviposition site, but in cages where the oviposition sites can be easily accessed by walking, this might be less important.

In Experiment 1, sawdust and coconut fiber at their minimum and maximum volumes produced the lowest and highest proportion of female fliers, respectively (Figure 1d). These results may be related to the capacity of substrates to absorb and dissipate heat [19]. Temperature is a critical factor during the first days of pupal development that can affect the flight ability of artificially reared *C. capitata* [18]. Furthermore, the tsl mutation of the *C. capitata* VIENNA 8 GSS make female flies susceptible to high temperatures [11]. It is possible that intermediate and high volumes of sawdust and coconut fiber promoted better and more stable temperatures than the other substrates, and this favored female survival and development. However, we recognize that future research is required to examine temperature changes in the substrate with larvae/pupae and to determine the optimal substrate temperature that maximizes pupation of this reproductive colony of *C. capitata* VIENNA 8 GSS. This should be done with higher volumes of larvae and substrates than the ones used in this study, to gain a clearer picture of the phenomenon at the mass-rearing level.

The moisture characteristics of pupation substrates is another critical issue in mass-rearing of tephritids [19,21]. Certainly, the moisture of pupation substrates is a factor that affects survival and performance in fruit flies [44,45,46,47,48]. A previous study indicated that coconut fiber with a higher moisture retention capacity than vermiculite, was advantageous for pupation of mass-reared *A. obliqua* [19]. In our study, pupation of *C. capitata* VIENNA 8 GSS in vermiculite outperformed pupation in coconut fiber (Figure 1a). Vermiculite was a much denser substrate with a lower moisture content and a lower moisture retention capacity than coconut fiber (Table 1). This may point to a preference of *C. capitata* to pupate in dry substrates [46]. In fact, pupation of *C. capitata* VIENNA 8 GSS in cellulose I with the lowest moisture content of all substrates (Table 1), was superior to pupation in substrates with a higher moisture content, such as coconut fiber (Figure 1a), fine wheat bran and sawdust (Figure 2a). Despite the high proportions of pupation obtained with cellulose I, one drawback of this substrate was that it was prone to clumping, hindering the flow of the pupal separation process. Fine wheat bran also clumps when moistened and some particles may attach to the pupating larvae (R. Aguilar personal observation). This was not observed with celluloses II and III in our study. Perhaps the fact that some substrates attach to larvae during pupation, could have affected pupal respiration with negative effects on adult emergence and flight ability as observed in our study (Figure 2c,d), but this needs to be confirmed in future studies.

The pupal weight of *C. capitata* varies significantly depending on whether the larvae pupate in vermiculite or pupate in the absence of any substrate [21]. This may be related to substrates preventing larvae from losing water during the first days of pupation [20]. In our study, the type of pupation substrate was not a predictor of female pupal weight of *C. capitata* VIENNA 8 GSS (Figure 2b). This may suggest that even the lowest volumes of substrate we used help larvae to prevent water loss.

The moments prior to and during pupation are critical for the insect’s wellbeing [20,40,42], and our study on pupation substrates addressed this aspect in the mass rearing of fruit flies for SIT applications. Even though mass-rearing processes tend to be unnatural, a current concept in insect rearing systems is that the rearing conditions should simulate as much as possible the natural conditions of the target insect [49]. Using a substrate for pupation for the reproductive colony of *C. capitata* VIENNA 8 GSS better simulates the natural conditions of this fly pest and this could in turn translate into a better quality of the flies used for SIT purposes. This should be tested in future studies by examining transgenerational effects of pupating in the absence of a substrate vs. pupating in a substrate on sexual performance of male flies.

We note that the type of pupation substrate to be used will be influenced by the availability of materials with stable quality, and that among the substrates we tested only fine wheat bran and sawdust were from local suppliers in Chiapas. As mentioned previously, sawdust produced the highest proportion of female pupae (Figure 1b). However, we note that sawdust was the only substrate that required processing prior to its use (i.e., it was sifted to remove chip pieces). A substrate that requires additional handling for its use is not ideal in a mass-rearing context, and for this reason, we did not include other substrates in our study such as sand because in our experience sand usually comes with impurities requiring cleaning prior to its use. In addition, certain types of sand can be abrasive to the larvae (their skin) and sand does not retain water well requiring constant re-moisturizing. Therefore, substrates that do not require any special processing for its use such as the cellulose substrates used here are more practical in a mass-rearing context.

Our study highlights the need to improve our understanding of the mechanisms by which substrates affect pupation and quality parameters of flies. Future studies should consider adopting a multivariate approach to further understand how substrate characteristics interact with environmental conditions (e.g., temperature and humidity) of the pupation rooms to influence fly pupation and other traits of flies of relevance for SIT applications, such as the time to emergence. A cost-effectiveness optimization perspective [50,51] could be useful to model how many times a single batch of substrate (e.g., cellulose III) could be reused, and how this translates into cost savings in the production process.

## 5. Conclusions

We conclude that cellulose III at relatively low volumes was an ideal pupation substrate for a reproductive colony of *C. capitata* VIENNA 8 GSS in terms of pupation, female pupal weight, female fliers, and estimated cost to produce one million pupae compared to the fine wheat bran substrate currently used in the Moscamed facility. Sawdust was more economical than cellulose III to produce one million pupae with a higher proportion of female pupae, which is very important, but with the drawback that it requires additional handling and has a potential cost associated with larval loss during pupation. We believe that both cellulose III and sawdust have potential for further testing at a larger scale considering other variables associated with production costs such as the recovery of female flies, the larvae that do not pupate and the possibility of recycling substrates. The study of pupation substrates is an issue that could improve the mass-rearing process of fruit flies for SIT applications by enhancing fly pupation and quality, and reducing substrate-associated costs.

## Figures and Tables

**Table 1:** The pupation substrates tested with their cost per kg and physicochemical characteristics. Pupation substrates are ordered from the lowest to highest cost per kg.

Pupation Substrate	Cost per kg (US $) ^1^	Density (g/cm^3^) ^2^	Moisture (%) ^2^	pH ^2^	Moisture Retention Capacity (%) ^2^	Granularity (Size Range)	Origin
Coconut fiber ^3^	0.36	0.112 (0.007)	13.0 (0.05)	4.5 (0.01)	804.6 (4.3)	149–1410 µm	Coconut shell
Fine wheat bran ^4^	0.49	0.413 (0.003)	11.2 (0.6)	6.3 (0.03)	456.5 (11.5)	149–1000 µm	Wheat husk
Vermiculite ^5^	0.94	0.254 (0.001)	6.8 (0.4)	5.8 (0.09)	378.7 (12.6)	149–1410 µm	Mineral
Sawdust ^6^	0.99	0.191 (0.018)	10.1 (0.2)	4.3 (0.05)	422.1 (3.7)	149–1410 µm	Wood
Cellulose III ^7^	2.00	0.206 (0.014)	8.8 (0.1)	4.6 (0.14)	560.2 (2.2)	74–149 µm	Wood dust
Cellulose II ^8^	2.10	0.266 (0.029)	8.2 (0.1)	4.4 (0.12)	500.4 (5.9)	74–149 µm	Wood dust
Cellulose I ^9^	2.10	0.080 (0.0004)	5.0 (0.2)	7.6 (0.22)	832.9 (4.2)	32–200 µm	Recycled paper

^1^ Exchange rate at the time of writing was US $1 = $20.20 Mexican pesos. In the case of fine wheat bran, vermiculite, coconut fiber and celluloses, wholesale costs with delivery at Moscamed are provided; in the case of sawdust, retail cost from local supplier is provided. ^2^ Mean values (standard deviation) (*n* = 3). ^3^ CoirTech, Coirtech, S.A. de C.V., Tecomán, Colima, Mexico. ^4^ Harinera de Chiapas, Harinera de Chiapas, S.A. de C.V., Arriaga, Chiapas, Mexico. ^5^ Radical, DISA Distribuidora de Sustratos Agrícolas SA de CV, Mexico. ^6^ Aserrín Multiusos, Productos en Madera S.A. de C.V., The Home Depot, Tapachula, Chiapas, Mexico. ^7^ Filtracel EFC 1100 T, Rettermaier Mexicana, JRS/Derivados Macroquímicos S.A de C.V., Ecatepec, Edo. Mex., Mexico. ^8^ Filtracel EFC 900 T, Rettermaier Mexicana, JRS/Derivados Macroquímicos S.A de C.V., Ecatepec, Edo. Mex., Mexico. ^9^ Arbocel, Rettermaier Mexicana, JRS/Derivados Macroquímicos S.A de C.V., Ecatepec, Edo. Mex., Mexico.

**Table 2:** Estimated quantities of third instar *C. capitata* VIENNA 8 GSS larvae and cost of pupation substrates at selected volumes required to produce one million pupae. Pupation substrates are listed from the lowest to highest cost for one million pupae.

Pupation Substrate	Substrate Volume (mL of Substrate/5 mL of Larvae) ^1^	Predicted Pupation (Proportion) ^2^	No. of Larvae Required to Produce 1 Million Pupae ^3^	Substrate Volume Required to Produce 1 Million Pupae (mL) ^4^	Weight of Substrate Volume Needed to Produce 1 Million Pupae (kg) ^5^	Substrate Cost for 1 Million Pupae (US $) ^6^
Cellulose I	2.5	0.9860	1,014,238	9486	0.76	1.59
Coconut fiber	12.5	0.8804	1,135,809	53,115	5.95	2.14
Sawdust	5.0	0.9623	1,039,204	19,439	3.71	3.67
Cellulose III	2.5	0.9860	1,014,238	9486	1.95	3.91
Fine wheat bran	5.0	0.9526	1,049,723	19,636	8.11	3.97
Vermiculite	5.0	0.9460	1,057,033	19,772	5.02	4.72
Cellulose II	2.5	0.9860	1,014,238	9486	2.52	5.30

^1^ The substrate volumes of 2.5, 5.0 and 12.5 mL/5 mL of larvae, are equivalent to substrate:larvae ratios of 0.5:1, 1:1 and 2.5:1, respectively. ^2^ Based on the GLM quasibinomial fitted to data on pupation from Experiment 1. In the case of celluloses II and III, we used the prediction of cellulose I. ^3^ One million pupae/predicted proportion of pupation. ^4^ [(No. of larvae required to produce 1 million pupae × 5 mL of larvae)/267.3 larvae in 5 mL] × Selected substrate volume in mL/5 mL of larvae. ^5^ Substrate density in g/cm^3^ × Substrate volume in mL required to produce 1 million pupae/1000 g. ^6^ Weight of substrate volume needed to produce 1 million pupae (kg) × Cost of substrate per kg in US$.

## Data Availability

Experimental data are available on request from the corresponding authors.

## Supplementary Materials

The following are available online at https://www.mdpi.com/article/10.3390/insects12040337/s1, Supplementary File S1: Linear predictor of the models fitted to the data of Experiment 1. Supplementary File S2: Multiple comparisons of means Experiment 2.
