# Supplementary material for: Pupation Substrate Type and Volume Affect Pupation, Quality Parameters and Production Costs of a Reproductive Colony of Ceratitis capitata (Diptera: Tephritidae) VIENNA 8 Genetic Sexing Strain
Source: Insects. 2021 Apr 9;12(4):337. doi: 10.3390/insects12040337 (PMC8069380; doi:10.3390/insects12040337)
Supplement: Supplementary file 1 [file insects-12-00337-s001.pdf]

insects

MDPI

Article

Pupation Substrate Type and Volume Affect Pupation, Quality Parameters and Production Costs of a Reproductive Colony of *Ceratitis capitata* (Diptera: Tephritidae) VIENNA 8 Genetic Sexing Strain

Carlos Pascacio-Villafán [1,*], Luis Quintero-Fong [2,*,†], Larissa Guillén [1], José Pedro Rivera-Ciprian [2], Reynaldo Aguilar [2] and Martín Aluja [1]

Supplementary File S1

Linear predictors of the Generalized Linear Models fitted to the data of the different response variables of Experiment 1 (See the Materials and Methods and Results section of the main text for details). We present the output from the analyses computed in the R software (R Development Core Team 2017).

Pupation (YBIN = a binary vector with data of larvae that pupated and larvae that do not pupated)

```
Call:
glm(formula = YBIN ~ SubstrateType + SubstrateVolumemL, family = quasibinomial)

Deviance Residuals:
    Min       1Q   Median       3Q      Max
-3.1348  -0.8115   0.0089   0.6253   3.7821

Coefficients:
                           Estimate Std. Error t value Pr(>|t|)
(Intercept)                 2.82200    0.15180  18.591  < 2e-16 ***
SubstrateTypeBSawdust       0.23769    0.17351   1.370   0.1777
SubstrateTypeCCellulose     1.34021    0.25091   5.341  3.1e-06 ***
SubstrateTypeDCoconutFiber -1.27371    0.13743  -9.268  6.6e-12 ***
SubstrateTypeEVermiculite  -0.13716    0.16594  -0.827   0.4129
SubstrateVolumemL           0.03586    0.01408   2.547   0.0144 *
---
Signif. codes:  0 '***' 0.001 '**' 0.01 '*' 0.05 '.' 0.1 ' ' 1

(Dispersion parameter for quasibinomial family taken to be 1.617764)

    Null deviance: 504.801  on 49  degrees of freedom
Residual deviance:  74.482  on 44  degrees of freedom
AIC: NA

Number of Fisher Scoring iterations: 4
```

Pupal sex ratio (YBIN = a binary vector with data of female pupae and male pupae)

```
Call:
glm(formula = YBIN ~ SubstrateType, family = quasibinomial)

Deviance Residuals:
    Min       1Q   Median       3Q      Max
-3.4923  -0.9731   0.1880   1.0014   5.2794

Coefficients:
                             Estimate Std. Error t value Pr(>|t|)
(Intercept)                 0.7162696  0.0702584  10.195 4.79e-13 ***
SubstrateTypeBSawdust       0.2437128  0.1018546   2.393   0.0212 *
SubstrateTypeCCellulose    -0.0007678  0.1027132  -0.007   0.9941
```

```
SubstrateTypeDCoconutFiber  -0.1798137  0.1101688  -1.632   0.1099
SubstrateTypeEVermiculite   -0.0817985  0.1040338  -0.786   0.4360
---
Signif. codes:  0 '***' 0.001 '**' 0.01 '*' 0.05 '.' 0.1 ' ' 1

(Dispersion parameter for quasibinomial family taken to be 2.835261)

    Null deviance: 171.97  on 47  degrees of freedom
Residual deviance: 124.46  on 43  degrees of freedom
  (2 observations deleted due to missingness)
AIC: NA

Number of Fisher Scoring iterations: 4
```

Female emergence (YBIN = a binary vector with data of females that emerged and females that did not emerged)

```
Call:
glm(formula = YBIN ~ 1, family = quasibinomial)

Deviance Residuals:
    Min       1Q   Median       3Q      Max
-4.6951  -0.6311   0.3362   0.7451   2.3971

Coefficients:
            Estimate Std. Error t value Pr(>|t|)
(Intercept)  0.13245    0.04265   3.106  0.00321 **
---
Signif. codes:  0 '***' 0.001 '**' 0.01 '*' 0.05 '.' 0.1 ' ' 1

(Dispersion parameter for quasibinomial family taken to be 1.458164)

    Null deviance: 69.474  on 47  degrees of freedom
Residual deviance: 69.474  on 47  degrees of freedom
  (2 observations deleted due to missingness)
AIC: NA

Number of Fisher Scoring iterations: 3
```

Female fliers (YBIN = a binary vector with data of females fliers and females that did not fly)

```
Call:
glm(formula = YBIN ~ SubstrateType * SubstrateVolumemL, family = quasibinomial)

Deviance Residuals:
    Min       1Q   Median       3Q      Max
-3.3653  -0.6650   0.1023   0.8286   2.1030

Coefficients:
                                        Estimate Std. Error t value Pr(>|t|)
(Intercept)                             0.098361   0.213536   0.461   0.6477
SubstrateTypeBSawdust                  -0.392161   0.292350  -1.341   0.1877
SubstrateTypeCCellulose                -0.146776   0.328765  -0.446   0.6578
SubstrateTypeDCoconutFiber             -0.536480   0.305119  -1.758   0.0868 .
SubstrateTypeEVermiculite               0.186829   0.303052   0.616   0.5412
SubstrateVolumemL                      -0.003939   0.025600  -0.154   0.8785
SubstrateTypeBSawdust:SubstrateVolumemL       0.053564   0.035396   1.513   0.1385
SubstrateTypeCCellulose:SubstrateVolumemL    -0.029018   0.038726  -0.749   0.4583
SubstrateTypeDCoconutFiber:SubstrateVolumemL  0.058439   0.037032   1.578   0.1228
SubstrateTypeEVermiculite:SubstrateVolumemL  -0.038669   0.036496  -1.060   0.2960
---
Signif. codes:  0 '***' 0.001 '**' 0.01 '*' 0.05 '.' 0.1 ' ' 1

(Dispersion parameter for quasibinomial family taken to be 1.361853)

    Null deviance: 84.906  on 47  degrees of freedom
Residual deviance: 52.934  on 38  degrees of freedom
  (2 observations deleted due to missingness)
AIC: NA
```

```
Number of Fisher Scoring iterations: 4
```

References

R Development Core Team. *R: A Language and Environment for Statistical Computing*; R Foundation for Statistical Computing: Vienna, Austria, 2017.

Supplementary File S2

This Supplementary File S2 presents the multiple comparisons of means performed after ANOVA tests detected significant effects of the predictor variable (i.e., substrate type) in the response variables (i.e., pupation, female emergence and female fliers) (See the Materials and Methods and Results section of the main text for details). We present the output of the Tukey contrasts computed in the R software (R Development Core Team 2017) using the *glht* function of the package *multcomp* (Hothorn et al. 2008).

Pupation

```
 Simultaneous Tests for General Linear Hypotheses

Multiple Comparisons of Means: Tukey Contrasts


Fit: lm(formula = logit(Pupation) ~ SubstrateType + Block)

Linear Hypotheses:
                              Estimate Std. Error t value Pr(>|t|)
Cellulose1 - Sawdust == 0       0.43584   0.17761   2.454   0.1137
Cellulose2 - Sawdust == 0       0.38489   0.17761   2.167   0.2047
Cellulose3 - Sawdust == 0       0.37152   0.17761   2.092   0.2356
FineWheatBran - Sawdust == 0   -0.09877   0.17761  -0.556   0.9808
Cellulose2 - Cellulose1 == 0   -0.05095   0.17761  -0.287   0.9985
Cellulose3 - Cellulose1 == 0   -0.06432   0.17761  -0.362   0.9962
FineWheatBran - Cellulose1 == 0 -0.53461   0.17761  -3.010   0.0291 *
Cellulose3 - Cellulose2 == 0   -0.01337   0.17761  -0.075   1.0000
FineWheatBran - Cellulose2 == 0 -0.48366   0.17761  -2.723   0.0608 .
FineWheatBran - Cellulose3 == 0 -0.47029   0.17761  -2.648   0.0729 .
---
Signif. codes:  0 '***' 0.001 '**' 0.01 '*' 0.05 '.' 0.1 ' ' 1
(Adjusted p values reported -- single-step method)
```

Female emergence

```
      Simultaneous Tests for General Linear Hypotheses

Multiple Comparisons of Means: Tukey Contrasts


Fit: lm(formula = logit(Emergence) ~ SubstrateType + Block)

Linear Hypotheses:
                              Estimate Std. Error t value Pr(>|t|)
Cellulose1 - Sawdust == 0      -0.52254   0.17806  -2.935   0.0356 *
Cellulose2 - Sawdust == 0      -0.31129   0.17806  -1.748   0.4121
Cellulose3 - Sawdust == 0      -0.19470   0.17806  -1.093   0.8093
FineWheatBran - Sawdust == 0   -0.46414   0.17806  -2.607   0.0805 .
Cellulose2 - Cellulose1 == 0    0.21126   0.17806   1.186   0.7591
Cellulose3 - Cellulose1 == 0    0.32785   0.17806   1.841   0.3590
FineWheatBran - Cellulose1 == 0  0.05841   0.17806   0.328   0.9974
```

```
Cellulose3 - Cellulose2 == 0    0.11659   0.17806  0.655  0.9652
FineWheatBran - Celulosa2 == 0  -0.15285   0.17806  -0.858  0.9109
FineWheatBran - Celulosa3 == 0  -0.26944   0.17806  -1.513  0.5576
---
Signif. codes:  0 '***' 0.001 '**' 0.01 '*' 0.05 '.' 0.1 ' ' 1
(Adjusted p values reported -- single-step method)
```

Female fliers

```
     Simultaneous Tests for General Linear Hypotheses

Multiple Comparisons of Means: Tukey Contrasts


Fit: lm(formula = logit(Voladoras...) ~ Sustrato + Block)

Linear Hypotheses:
                          Estimate Std. Error t value Pr(>|t|)
Cellulose1 - Sawdust == 0     -0.44476   0.12312  -3.612  0.0051 **
Cellulose2 - Sawdust == 0     -0.27115   0.12312  -2.202  0.1913
Cellulose3 - Sawdust == 0     -0.11537   0.12312  -0.937  0.8814
FineWheatBran - Sawdust == 0   -0.34400   0.12312  -2.794  0.0510 .
Cellulose2 - Cellulose1 == 0    0.17361   0.12312   1.410  0.6234
Cellulose3 - Cellulose1 == 0    0.32939   0.12312   2.675  0.0684 .
FineWheatBran - Cellulose1 == 0  0.10076   0.12312   0.818  0.9241
Cellulose3 - Cellulose2 == 0    0.15578   0.12312   1.265  0.7131
FineWheatBran - Cellulose2 == 0 -0.07285   0.12312  -0.592  0.9759
FineWheatBran - Cellulose3 == 0 -0.22863   0.12312  -1.857  0.3502
---
Signif. codes:  0 '***' 0.001 '**' 0.01 '*' 0.05 '.' 0.1 ' ' 1
(Adjusted p values reported -- single-step method)
```

References

Hothorn, T.; Bretz, F.; Westfall, P. Simultaneous inference in general parametric models. *Biom. J.* **2008**, *50*, 346–363.

R Development Core Team. *R: A Language and Environment for Statistical Computing*; R Foundation for Statistical Computing: Vienna, Austria, 2017.
